# Biallelic variants in SREK1 downregulating SNORD115 and SNORD116 cause a Prader-Willi–like syndrome

**DOI:** 10.1172/JCI191008

**Published:** 2025-06-22

**Authors:** Sadia Saeed, Anna-Maria Siegert, Y.C. Loraine Tung, Roohia Khanam, Qasim M. Janjua, Jaida Manzoor, Mehdi Derhourhi, Bénédicte Toussaint, Brian Y.H. Lam, Sherine Awad Mahmoud, Emmanuel Vaillant, Emmanuel Buse Falay, Souhila Amanzougarene, Hina Ayesha, Waqas I. Khan, Nosheen Ramazan, Vladimir Saudek, Stephen O’Rahilly, Anthony P. Goldstone, Muhammad Arslan, Amélie Bonnefond, Philippe Froguel, Giles S.H. Yeo

**Affiliations:** 1 INSERM UMR 1283, CNRS UMR 8199, European Genomic Institute for Diabetes, Institut Pasteur de Lille, Lille, France.; 2 University of Lille, Lille University Hospital, Lille, France.; 3 Department of Metabolism, Digestion and Reproduction, Imperial College London, London, United Kingdom.; 4 Medical Research Council Metabolic Diseases Unit, Institute of Metabolic Science, University of Cambridge, Cambridge, United Kingdom.; 5 KAM School of Life Sciences, Forman Christian College, Lahore, Pakistan.; 6 College of Medicine and Health Sciences, National University of Science and Technology, Sohar, Oman.; 7 Department of Paediatrics, Punjab Medical College, Faisalabad, Pakistan.; 8 The Children’s Hospital and Institute of Child Health, Multan, Pakistan.; 9 Department of Special Education, Punjab, Pakistan.; 10 PsychoNeuroEndocrinology Research Group, Division of Psychiatry, Department of Brain Sciences, Faculty of Medicine, Imperial College London, London, United Kingdom.

**Keywords:** Cell biology, Genetics, Neuroscience, Molecular genetics, Monogenic diseases, Obesity

## Abstract

Biallelic variations in *SREK1* reduce *SNORD115*/*116* expression, linking severe obesity and Prader-Willi-like traits, offering genetic and molecular insights into a new form of syndromic obesity.

**To the Editor:** Monogenic forms of severe, early-onset obesity are frequently linked to disruptions in the central leptin/melanocortin axis, a crucial appetite control pathway (1). Yet, syndromic obesity — marked by neurodevelopmental and/or behavioral features — suggests the involvement of additional molecular mechanisms. Prader-Willi syndrome (PWS) remains the best-known example, but emerging genetic studies have revealed other pathways (2). Studying individuals from consanguineous populations offers a powerful strategy to uncover rare, autosomal recessive causes of such complex phenotypes.

The Severe Obesity in Pakistani Population (SOPP) cohort (*n* = 463) is one such study. We performed burden analysis on whole-exome sequencing data using the mixed-effects score test (MiST) framework (3) and identified significant enrichment (Pπ = 0.021; π̂ = 14; Pτ = 1.0) of rare, homozygous, and potentially deleterious variants in serine/arginine-rich splicing factor kinase 1 (*SREK1*). Three unrelated families harbored distinct *SREK1* (NM_1323533) variants — c.284C>T (p.P95L), c.581C>T (p.T194M), and c.1801G>A (p.E601K) — which segregated with disease in a recessive pattern and were not observed in the homozygous state in population databases, including gnomAD version 4.0.0 (Figure 1A).

Structural modeling using AlphaFold suggested that p.P95L and p.T194M lie within each of 2 conserved RNA recognition motifs (RRMs) critical for splicing regulation, whereas p.E601K lies in a less-characterized C-terminal region (Figure 1B). To functionally characterize these variants, we introduced each into human induced pluripotent stem cells (iPSCs) via CRISPR/Cas9 and differentiated them into hypothalamic neurons (Supplemental Figure 1; supplemental material available online with this article; https://doi.org/10.1172/JCI191008DS1) — a physiologically relevant model previously used to study obesity-related variants.

Bulk RNA-Seq revealed that neurons harboring RRM domain variants (p.P95L, p.T194M) clustered distinctly from both WT and p.E601K neurons (Supplemental Figure 2), with marked downregulation of several small nucleolar RNAs, notably *SNORD115* and *SNORD116* (Figure 1C). Formerly known as *HBII-52* and *HBII-85*, these short (~100 nt) noncoding RNAs were quantified by RT-PCR due to RNA-Seq detection limits. This confirmed a 50% downregulation of *SNORD115* and *SNORD116* expression in the RRM domain variants versus WT. In contrast, p.E601K showed no change (Figure 1D). Relative expression values (normalized to GAPDH): SNORD116a - WT: 1.15, P95L: 0.50, T194M: 0.30, E601K: 1.11; SNORD116b - WT: 1.24, P95L: 0.34, T194M: 0.37, E601K: 1.40; SNORD115 - WT: 1.23, P95L: 0.54, T194M: 0.25, E601K: 1.81. Given the role of SREK1 in splicing, these reductions likely reflect transcriptional downregulation rather than increased RNA turnover.

Deletions of the *SNORD116* cluster cause PWS (4), and our previous work showed that hypothalamus-specific deletion of *Snord116* in adult mice results in PWS-like hyperphagia (5). Thus, the downregulation of *SNORD115/116* associated with *SREK1* mutations prompted clinical evaluation for PWS-like traits.

All 3 probands exhibited childhood-onset obesity and varying developmental delays. The p.P95L proband was first evaluated at 8 years of age (BMI: 29 kg/m²), with the BMI rising to 48 kg/m² by 15.5 years of age, with hyperphagia, food-seeking behavior, delayed motor milestones, and hypotonia. The p.T194M proband presented at 19 years of age (BMI: 56 kg/m²), reporting lifelong compulsive eating and behavioral disturbances. The p.E601K proband was assessed at 11 years of age (BMI: 38 kg/m²), with the BMI rising to 41 kg/m² by age 14 and mild cognitive delay and social shyness, but not hyperphagia. Detailed clinical and anthropometric data are provided in Supplemental Tables 1 and 2.

To assess overlap with PWS, we used the Dykens Hyperphagia Questionnaire at follow-up (6). Both RRM domain probands scored within or above the PWS range (Supplemental Tables 1 and 2). The p.P95L proband met all major PWS criteria: infantile hypotonia, feeding difficulties, global developmental delay, characteristic facial features of a small mouth, a thin upper lip, an IQ of 49 and compulsive food-seeking. The p.T194M proband, although not meeting the criterion of obesity onset before age 6, gained substantial weight from age 9 and presented at 19 years of age with a BMI of 56 kg/m². He met multiple PWS traits including severe hyperphagia, food obsession, binge-eating, anxiety, depression, and social withdrawal. He had a low-normal IQ of 80 (16th percentile), academic difficulties, facial features consistent with PWS, hypertension, and sleep apnea. In contrast, the p.E601K proband lacked overt hyperphagia or clear PWS-like traits. Despite no formal neurocognitive testing because of a lack of consent, the absence of *SNORD115/116* dysregulation suggests that the p.E601K variant may be benign or at least of uncertain significance.

These findings show that *SREK1* RRM domain variants disrupted a critical noncoding RNA network regulating hypothalamic satiety, phenocopying aspects of PWS, and highlighting the relevance of this pathway in hyperphagia. This work demonstrates the power of consanguineous cohorts like the SOPP study cohort for identifying previously unrecognized rare, recessive causes of complex disease.

In conclusion, we discuss here a previously unreported cause of syndromic obesity, that of biallelic *SREK1* mutations in RRMs that impair *SNORD115/116* RNA expression and mimic aspects of PWS via a splicing-driven mechanism. This insight expands the genetic architecture of syndromic obesity and reveals a unique intersection between RNA splicing and noncoding RNA regulation in human metabolic disease.

## Supplementary Material

Supplemental data

Supporting data values

## Figures and Tables

**Figure 1:**
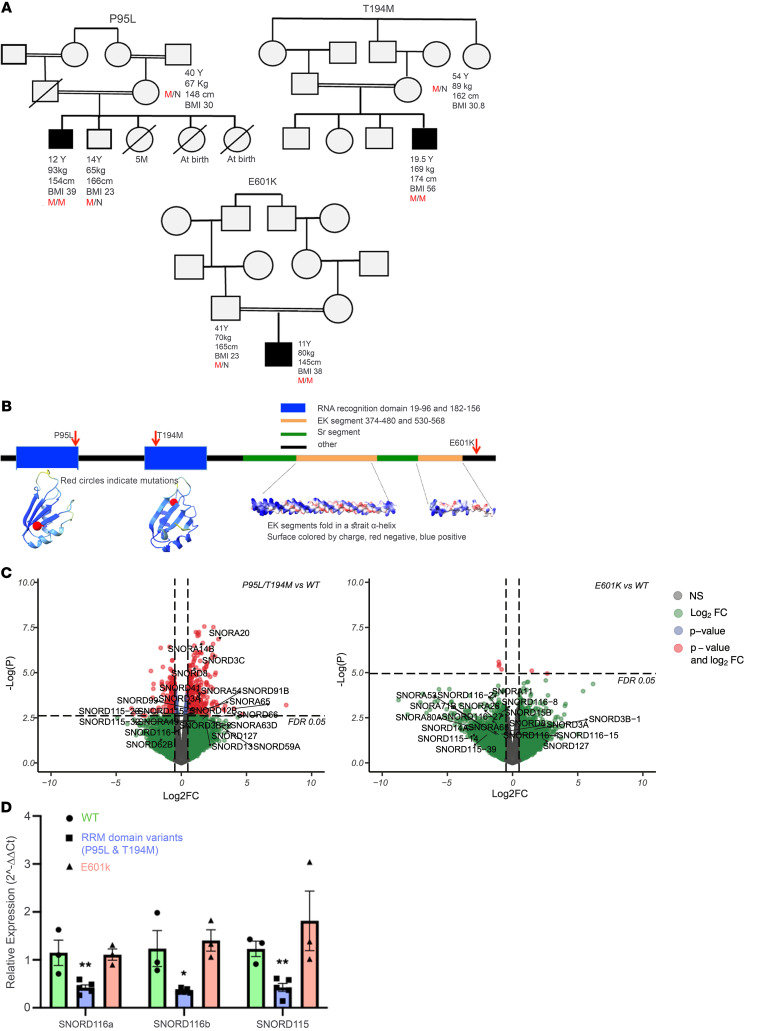
Rare biallelic SREK1 variants lead to downregulation of SNORD115 and SNORD116 and are associated with a syndromic obesity phenotype resembling Prader-Willi syndrome. (**A**) Pedigrees of 3 families with *SREK1* variants. Affected individuals are shown with shading. M/M and M/N indicate homozygosity and heterozygosity, respectively. (**B**) SREK1 domain structure and AlphaFold-predicted model highlighting RRMs and EK helices. (**C**) Volcano plots showing downregulation of SNORD115 and SNORD116 in RRM domain variants. Differential expression analysis of RRM variants versus WT revealed dysregulation of multiple small nucleolar RNAs, particularly in the SNORD115 and SNORD116 families. EdgeR’s quasi-likelihood F-test with a significance threshold of FDR < 0.05. FC, fold change. (**D**) Quantitative RT-PCR confirmed reduced SNORD115/116 expression in RRM variants versus WT and p.E601K. Multiple unpaired 2-tailed *t* test. Data indicate the mean ± SEM; *P < 0.05 and **P < 0.01.

## Version 1 06/23/2025

In-Press Preview

## Version 2 08/15/2025

Electronic publication
