# Supplementary material for: Biallelic variants in SREK1 downregulating SNORD115 and SNORD116 cause a Prader-Willi–like syndrome
Source: J Clin Invest. 2025 Jun 22;135(16):e191008. doi: 10.1172/JCI191008 (PMC12352886; doi:10.1172/JCI191008)
Supplement: Supplemental data [file jci-135-191008-s292.pdf]

Supplemental Materials

Biallelic variants in SREK1 downregulating SNORD115 and SNORD116 cause a Prader-Willi-like syndrome

Supplemental Figures

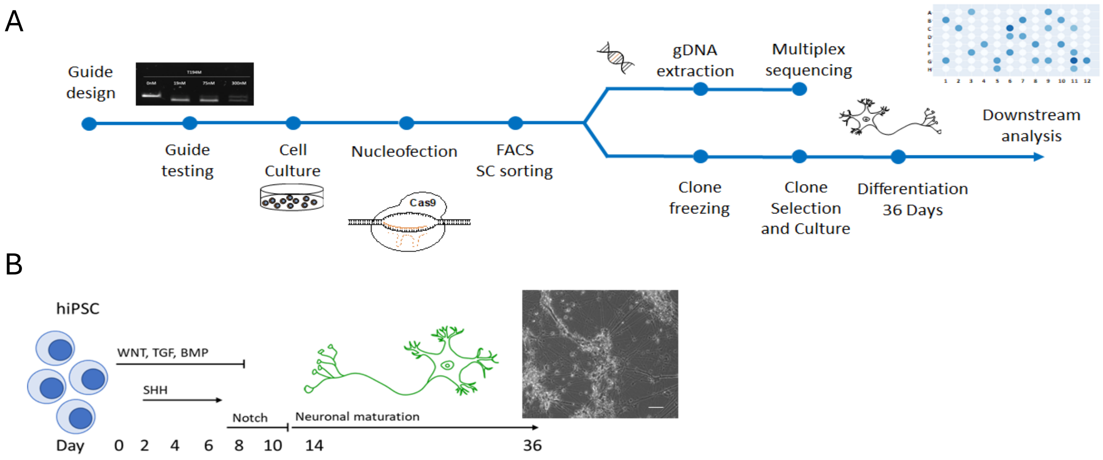

Figure S1: Experimental design of gene-editing pipeline

(A) The schematic outlines the experimental workflow, starting with *in vitro* guide RNA testing for targeted ssODN-mediated mutation knock-ins. Following CRISPR gene targeting, iPSC clones were isolated using FACS into 96-well plates, which were duplicated to support clone propagation and the barcoded PCR amplification of the *SREK1* locus to facilitate deep sequencing to identify clones harbouring the mutations of interest. **(B)** Selected clones, identified using GenEditID, were expanded and cryopreserved before undergoing a 36-day neuronal differentiation protocol. Downstream analysis was performed after FACS, which enriched neuronal populations using a combination of four cell surface marker antibodies.

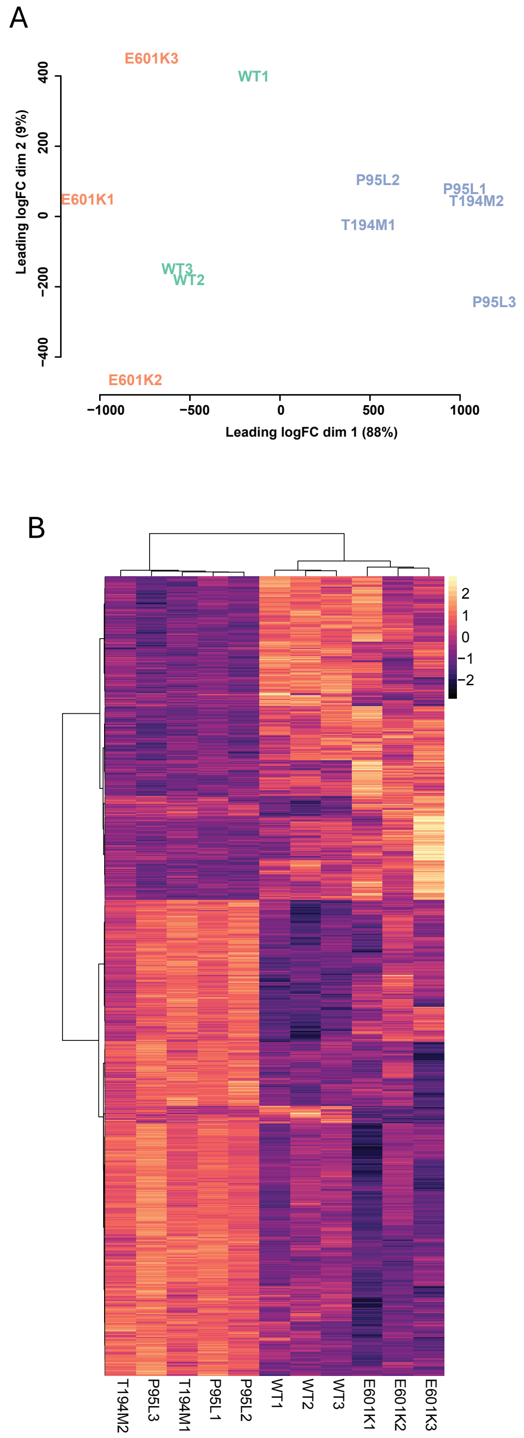

Figure S2: RNAseq results for *SREK1* RNA recognition domain and p.E601K variants

(A) Principal Component Analysis (PCA) plots of RNAseq data comparing wild-type (WT) *SREK1* and variants. Tight clustering within each genotype group reflects high reproductivity across replicates. Notably, the P95L and T194M variants cluster closely, suggesting shared downstream effects, while the E601K variant forms a distinct cluster, highlighting a potentially divergent biological impact. **(B)** Heatmap of differentially expressed genes (DEGs) that visualises the changes in gene expression associated with different variants in the *SREK1* gene. The colour intensity corresponds to the level of gene expression, with darker colours indicating stronger expression changes.

Supplemental Tables

Table S1: Integrated clinical, familial, cognitive, behavioral, and metabolic features in individuals with biallelic SREK1 variants (p.P95L and p.T194M)

Patient's characteristics	**p.*P95L***	**p.*T194M***
Age (y)	15.4	26.6
Sex	Male	Male
Height (m)	1.60	1.80
Weight at assessment (kg)	124	115
BMI at assessment (kg/m2)	48.4	35.5
Maximum adult weight (kg)	n/a	195
Age onset hyperphagia (y)	3	9
Dykens hyperphagia questionnaire		
Hyperphagia - total (11-55)	37	39
Hyperphagia - behaviour (5-25)	15	21
Hyperphagia - drive (4-20)	16	13
Hyperphagia - severity (2-10)	6	5
Variability hyperphagia (low 1, high 5)	2	1
PWS diagnostic criteria		
PWS major criteria (max 7)	5	4
PWS minor criteria (max 11)	4	4
PWS supportive criteria (max 8)	3	3
Missing major answers (max 7)	0	0
Missing minor answers (max 11)	0	0
Missing supportive answers (max 8)	2	2
Total Holm score (max. 12.5)	7	6
Max. possible Holm score as missing answers	12.5	12.5
PWS diagnostic criteria met (>6 total, >3 major)	Yes	No
Family history		
Consanguinity	Yes	Yes
Early-onset obesity	No	No
Diabetes mellitus	No	Mother, sister
Other	3 sisters died 6-7 months	Paternal nephew speech hearing problem
Cognitive testing (WAIS-R)		
Verbal IQ	69	88
Performance IQ	67	76
Full-scale IQ	49	80
IQ percentile	?	16.1
Overall IQ category	Intellectual disability	Below normal
Blood parameters		
Fasting glucose (mmol/L)	4.7	4.2
Fasting insulin (pmol/L)	42.9	119.4
HOMA-IR	1,28	3.18
HOMA-β (%)	106.3	518.7
HbA1c (%)	4.7	5.7

HbA1c (mmol/mol)	28	39
Fasting cholesterol (mmol/L)	3.52	2.82
eGFR (ml/min/1.73m2)	122	NA
Creatinine (umol/L)	75	69
Free thyroxine (child 11.6-18.0, adult 10.3-34.8 pmol/L)	11.5	13.3
TSH (0.4-4.5 mU/L)	3.68	4.25
ALT (<45 IU/L)	27	33
AST (<35 IU/L)	35	35
gGT (<59 IU/L)	10	28
Testosterone (14-16yo 1.2-21.9, adult 9.0-34.7 nmol/L)	15.0	4.9
Oestradiol (40-162 pmol/L)	132	103
LH (0.57-12.07 IU/L)	4.95	3.37
FSH (0.95-11.95 IU/L)	3.78	5.78
Major PWS Diagnostic Criteria	**p.P95L**	**p.T194M**
Neonatal hypotonia	Yes	Yes
Poor suck	Yes	Yes
Challenge feeding at birth	Yes	Yes
Poor weight gain in infancy	No	Yes
Obesity	Yes	Yes
Age onset (y)	1-5	5-9
Hyperphagia	Yes	Yes
Facial appearance		
Increased skull AP dimension	No	No
Narrow bifrontal diameter	No	Yes
Small mouth with thin upper lip	Yes	Yes
Down-turned corners mouth	Yes	No
Almond shaped palpebral fissure	No	No
Hypogonadism	No	No
Hypogenitalism	No	No
Learning disability	Yes	No
Global developmental delay <6y	Yes	Yes
Minor PWS Diagnostic Criteria	**p.P95L**	**p.T194M**
Lethargy as infant	Yes	Yes
Temper tantrums	Yes	Yes
Violent outbursts	Yes	Yes
Obsessive / compulsive behaviours	Yes	Yes
Tendency to be argumentative, oppositional, rigid, manipulative, possessive, stubborn	Yes	Yes
Perseverates	Yes	Yes
Steals	?	Yes
Lies	Yes	Yes
Disturbed or noisy sleep	Yes	No
Sleep apnoea	Yes	Yes
**Physical appearance**		
Short stature by 15y	No	No
Fair skin or hair	Yes	Yes

Small hands	No	No
Small feet	No	No
Narrow hands with straight ulnar border	No	No
Eye related issues		
Myopia	No	No
Esotropia (squint)	No	No
Thick saliva with crusting at mouth corners	No	No
Speech articulation defects	No	No
Skin picking	No	No
High pain threshold	Yes	Yes
Temperature dysregulation	Yes	Yes
Decreased vomiting	Yes	Yes
Depression	No	Yes
Anxiety	Yes	Yes
Psychosis	No	No
Compulsive counting/hoarding	No	Yes
Autism	No	No
Epilepsy	No	No
Undescended testes	No	No
Need for nasogastric feeding as newborn	No	No
Metabolic features		
Hypertension	No	Yes
Insulin resistance (HOMA-IR >2.5)	Yes	No
Acanthosis nigricans	Yes	No
Diabetes mellitus	No	No
Hypercholesterolaemia	No	No
Hypothyroidism	No	No
Hyponatraemia	No	No
Renal impairment	No	No
Fatty liver disease	No	No

Table S2: Comparative analysis of hyperphagia across biallelic *SREK1*, biallelic *LEPR* and heterozygous *MC4R* variants, and PWS, using Dykens Hyperphagia Questionnaire

Dykens Hyperphagia Questionnaire	*SREK1* p.P95L	*SREK1* p.T194M	*LEPR* (n=8)	*MC4R* (n=7)	PWS (n=21)	PWS (n=24)	PWS (n=21)	PWS (n=100)
**Reference no.**			(1)	(1)	(2)	(3)	(4)	(5)
**Age (years)**	15.4	26.6	7.1 ± 5.7	10.7 ± 6.2	15.5 ± 6.6	26.3 ± 6.9	27.8 ± 8.3	28.2 ± 7.7
**Sex**	Male	Male	Male & female	Male & female	Male & female	Male & female	Male & female	Male & female
**BMI at assessment (kg/m²)**	48.4	35.5	45.3 ± 15.9	35.1 ± 10.0	24.3 ± 6.8	30.2 ± 6.4	30.5 ± 6.3	42.0 ± 10.6
**Hyperphagia - behaviour (5-25)**	15	21	13.4 ± 3.9	11.9 ± 2.3	16.4 ± 3.9	14.1 ± 5.8	14.1 ± 5.2	12.8 ± 4.3
**Hyperphagia - drive (4-20)**	16	13	13.1 ± 3.6	14.0 ± 0.8	13.6 ± 3.7	11.2 ± 4.2	10.9 ± 3.7	11.5 ± 3.3
**Hyperphagia - severity (2-10)**	6	5	5.5 ± 2.9	5.1 ± 2.3	7.7 ± 1.5	4.5 ± 2.6	4.6 ± 2.7	4.6 ± 2.1
**Hyperphagia - total (11-55)**	37	39	32.0 ± 9.3	21.4 ± 5.5	39.7 ± 11.8	29.8 ± 10.1	29.6 ± 9.5	28.9 ± 8.0

Supplemental Methods

Sex as a biological variable

Our study examined male and female human, and similar findings are reported for both sexes.

Study population and genetic analysis

All probands with severe obesity from the SOPP cohort (*N*=550) were systematically screened for variants in *LEP* and *MC4R* genes through Sanger sequencing. The pathogenicity of these variants was evaluated in accordance with the guidelines and standards established by the American College of Medical Genetics and Genomics (ACMG) (6). Probands who tested negative for (likely) pathogenic variants in these two genes underwent further analysis through whole-exome sequencing, as detailed in previous studies (7). All genes known to be associated with monogenic (syndromic/non-syndromic) obesity were examined for (likely) pathogenic variants. In cases where we could not identify a potentially causative variant (n=463) in known monogenic (syndromic or non-syndromic) obesity genes, we utilized MiST analysis to uncover novel causative genes.

The MiST method was first published in 2013 (8) and since has been used in several studies that demonstrated that MiST performs best with regard to its statistical power across a range of architectures. Thus, for the next gene-centric analysis, we used the MiST method to identify a burden of variants significantly increasing the risk of obesity. This analysis included 463 patients with genetically unexplained obesity from the SOPP cohort and 1,000 controls from the Pakistani PROMIS (Pakistan Risk of Myocardial Infarction Study) population (9). Details of the analysis are provided elsewhere (10). Both participant groups originate from the same geographical region of Pakistan, facilitating a more accurate comparison. This approach enables the identification of genes that carry a significant burden of rare (i.e., minor allele frequency below 1%), homozygous, and potentially deleterious variants (as determined by SIFT and PolyPhen) among the obesity cases from the SOPP. Following the MiST analysis, additional filtration criteria were applied, including identification in three or more families, segregation of the variant with the disease within the family, and predominant expression of candidate genes in the human brain based on GTEx (11) and Hypomap data (12).

Structure Analysis of SREK1

The domain composition of the SREK1 protein is taken from InterPro database entry Q8WXA9. The structural model of the RRM domains is extracted from AlphaFold database, entry A0A2I2YTW4 (gorilla sequence, the human splice variant with 2 RRM domains is absent in UniProt and consequently also in AlphaFold).

Cell lines and routine cell culture

Human KOLF2.1J embryonic stem cells were cultured feeder-free on Geltrex (Thermo Fisher Scientific A1413202 in StemFlex medium (Thermo Fisher Scientific A3349401). Cells were passaged using TrypLE Express (Gibco) with 10 µM ROCK inhibitor (Y-27632, Stemcell Technologies 72304). Routine mycoplasma screening was performed using the EZ-PCR Mycoplasma Test Kit (Biological Industries 20-700-20) following the manufacturer's protocol.

CRISPR-Cas9-mediated targeting of SREK1

Three guide RNAs were designed using the Broad Institute's CRISPick tool (https://portals.broadinstitute.org/gppx/crispick/public), selected based on proximity to the target site, predicted on-target activity, and minimal off-target potential. For guide RNA synthesis, 120-nt oligos containing an SP6 promoter, guide sequence, and scaffold were transcribed in vitro using the MEGAscript SP6 kit (Thermo Fisher, AM1330). Guide efficiency was assessed in vitro, and the most active guide was selected for iPSC transfection. HDR templates were 100-nt single-stranded oligodeoxynucleotides (ssODNs), designed using IDT's Alt-R HDR tool (https://www.idtdna.com/pages/tools/alt-r-crispr-hdr-design-tool), containing the target variant and PAM-disrupting silent mutations to prevent re-cutting.

CRISPR-Cas9 ribonucleoprotein (RNP) complex-mediated editing in iPSCs

CRISPR RNPs were formed by incubating 3 µg sgRNA with 4 µg Cas9 (IDT) for 45 minutes. The RNPs and 1 µl of 100 µM ssODN were electroporated into 2×10^5 KOLF2.1J cells using the Amaxa 4D-Nucleofector™ with program CA137. Transfected cells were plated on Geltrex-coated plates in

StemFlex medium with Revitacell and Pen/Strep, and 30 µM HDR enhancer (IDT) was added. Cells were cultured under cold shock conditions (32°C, 5% CO_2) for 48 h to improve HDR efficiency and allowed to recover for 5–6 days. Single-cell clones were sorted into Geltrex-coated 96-well plates using an Aria-Fusion sorter. After ~2 weeks, viable colonies were expanded into duplicate plates for cryopreservation and gDNA extraction. Target regions were amplified by PCR (150–200 bp) using Fluidigm-linked primers, followed by indexing PCR for sample barcoding. Libraries were pooled based on band intensity, purified with Ampure XP beads, and quantified via Agilent Bioanalyzer. Sequencing was performed on a MiSeq Nano platform (CRUK Genomics Core). Genotypes were identified using the GenEditID workflow (https://genedotod/gotjib/op/). Karyotype analysis of selected clones was performed via G-banding by the University of Cambridge Core Laboratory at Stemnovate Ltd. The overall gene-editing and analysis workflow is outlined in Supplemental Figure S1.

Hypothalamic neuron differentiation of iPSCs carrying SREK1 variant of interest

To promote reproducible and mature hypothalamic neuron differentiation, we adapted a chemically defined protocol based on previously published methods (13-15). iPSCs carrying SREK1 variants were maintained in antibiotic-free StemFlex medium supplemented with 10 µM ROCK inhibitor (Y-27632) and cultured overnight on Geltrex-coated 10 cm dishes. The following day, neuroectodermal induction was initiated using dual SMAD inhibition (LDN193289, SB431542) and Wnt signaling inhibition (XAV939) in in-house N2B27-based neural induction medium. From days 2-7, ventral diencephalic patterning was achieved by activating Sonic Hedgehog signaling with 1 µM SAG and 1 µM purmorphamine, while gradually withdrawing SMAD/Wnt inhibitors. Media were refreshed every two days. At day 8, cells were transitioned to N2B27 supplemented with 5 µM DAPT to promote cell cycle exit. On day 14, cells were enzymatically dissociated using TrypLE and papain (Worthington), and re-plated onto laminin-coated 6-well plates (3×10^6 cells/well) in N2B27 supplemented with 10 ng/ml BDNF to initiate neuronal maturation. From day 16, media were replaced with Synaptojuice 1 (N2B27 + 10 ng/ml BDNF, 2 µM PD0332991, 5 µM DAPT, 370 µM $CaCl_2$, 1 µM LM22A4, 2 µM CHIR99021, 300 µM GABA, and 10 µM NKH477). After 7 days, cells were transitioned to Synaptojuice 2 (N2B27 +

10 ng/ml BDNF, 2 µM PD0332991, 370 µM $CaCl_2$, 1 µM LM22A4, 2 µM CHIR99021) and maintained until day 36. Media were refreshed every other day throughout differentiation and maturation.

Fluorescence-activated cell sorting of neurons

To isolate post-mitotic neurons from differentiated cultures, we applied fluorescence-activated cell sorting (FACS) based on established surface marker signatures (16). At day 28 of differentiation, cells were dissociated and resuspended in FACS buffer (PBS with 1% BSA and 2 mM EDTA), then filtered through 5 ml polystyrene tubes with cell-strainer caps (Falcon). Using a panel of four surface markers, we enriched for neurons defined as CD184−/CD44−/CD15LOW/CD24+, effectively separating them from progenitor and glial populations. Sorting was performed on a BD FACS Aria II cytometer (BD Biosciences).

Differential gene expression analysis

Total RNA was extracted from differentiated hypothalamic neurons using the Qiagen RNeasy Plus Micro Kit. RNA integrity was verified with the Agilent 2100 Bioanalyzer. Libraries were prepared using the Takara SMARTer Stranded Total RNA-Seq Kit v2, multiplexed, and sequenced on an Illumina NovaSeq 6000 platform (≥30 million reads/sample). Reads were aligned to the GRCh38 reference genome (Ensembl v100) using STAR (v2.5.0a), and differential gene expression was assessed using EdgeR's quasi-likelihood F-test with FDR < 0.05. Raw sequencing data are available at NCBI GEO (GSE292889).

Quantitative Real Time PCR

Ct values across samples and significance were determined using the multiple unpaired *t*-test. Expression levels of SNORD115 and SNORD116 (Groups I and II) were validated using quantitative real-time PCR (qPCR), with primers designed as previously described (17). Total RNA was reverse transcribed in triplicate using SuperScript™ IV Reverse Transcriptase (Invitrogen, 18090050), and qPCR was performed using SYBR™ Green Master Mix (Applied Biosystems, 4309155) on a QuantStudio 5 system. The thermal profile included an initial denaturation at 95°C for 15 min, followed by 45 cycles of 95°C for 15 s and 60°C for 30 s. GAPDH was used as the internal control for normalization.

Detailed clinical assessment on hyperphagia and PWS

Hyperphagia was assessed using the hyperphagia questionnaire from Dykens et al., which was originally designed and validated for patients with PWS to assess their specific food-related preoccupations and problems (18). The physician completed the questionnaire by asking questions to the parents and/or the proband. The Dykens Hyperphagia Questionnaire includes 13 items, each rated on a 5-point Likert scale where "0" indicates "not a problem" and "5" indicates a "severe/frequent problem." Three sub-scores are calculated: hyperphagic behavior, food-seeking behavior, and food-related preoccupations. Clinical diagnostic criteria for PWS used that devised by Holm et al (19). There are three categories of diagnostic criteria for PWS: major, minor and supportive, and these are scored on a weighted point system. Major criteria are valued at one point and minor at one half point. Supportive criteria are not included in the point system but serve to increase the confidence in the results determined by the other criteria. In children 3 years of age and younger, only 5 points are required for diagnosis, four of those must come from the major group. A total score of eight is necessary for the diagnosis in the 3 years to adulthood group. In this age group, major criteria items must comprise five or more points of the total score.

Proband detailed biochemical analysis

Metabolic markers, including plasma/serum leptin and insulin were measured using commercially available ELISA kits from Monobind (Lake Forest, USA). Blood glucose, HbA1c, lipids (cholesterol and triglycerides), markers for thyroid function (free thyroxine, TSH), gonadal function (testosterone, oestradiol, LH, FSH), renal function (electrolytes), and liver function (ALT, AST, gGT) were determined using commercial chemistry analysers.

Statistics

For statistical analyses, GraphPad Prism 7, Microsoft Excel, and R were used. All data involving a statistical analysis being reported met the criteria to use the appropriate statistical tests. Statistical tests used are reported in the figure legends. Variant calling was performed using the Genome Analysis Toolkit (GATK) following best practices. Variants were filtered based on depth of coverage (>20x) and

Phred-scaled quality scores (>30). Annotation was conducted using Ensembl Variant Effect Predictor (VEP), with population allele frequencies obtained from gnomAD and ClinVar for pathogenicity assessment. Differential gene expression analysis, if applicable, was conducted using EdgeR's Quasi-likelihood F-test with a significance threshold of False Discovery Rate (FDR) < 0.05. Gene expression was quantified using the comparative ΔΔCt method. Statistical significance was assessed using multiple unpaired, two-tailed Student's t-tests for pairwise comparisons. To confirm overall group differences, a non-parametric alternative to one-way ANOVA, the Kruskal–Wallis test, was also performed, followed by Dunn's post hoc test for multiple comparisons. Significance thresholds were set at $p < 0.05$ and $p < 0.01$, as indicated in the figure legends.

Conflict-of-interest statement

The authors have declared that no conflict of interest exists

Study approval

The clinical studies were approved by the institutional and hospital ethical committees, and written informed consent was obtained from all study participants or their legal guardians. All procedures involving human subjects adhered to the Declaration of Helsinki.

Data availability

The data presented in Figure 1D are available in the Supporting Data file online. Raw RNA sequencing data are available at NCBI GEO under accession number GSE292889. Whole-exome sequencing (WES) data are available with a transfer agreement.

Authors Contribution

SS, AB, PF, YCLT and GSHY conceptualized and designed the study. Methodology was developed by SS, AMS, YCLT, AB, PF, and GSHY. RK, QMJ, JM, HA, WIK, and MA were responsible for recruiting participants, as well as preparing and collecting the data. Experiments and formal data analysis were conducted by SS, AMS, YCLT, MD, BT, BYHL, SA, EV, EBF, SA, and AB. VS performed the protein modelling experiments. NR conducted detailed psychological analysis of the patients. APG contributed by comparing data with PWS patients and provided the necessary forms for this comparison. SOR

provided expert clinical insights on the diagnosis and data interpretation. The first draft of the manuscript was written by SS and YCLT. The manuscript was finalized collaboratively by SS, YCLT, SO, AB, PF, and GSHY. All authors reviewed, revised, and approved the final draft. SS, AMS, and YCLT are co–first authors and contributed equally to this work; the order of authorship among them was determined by mutual agreement.

Acknowledgements

We thank Frédéric Allegaert, Timothée Beke and Stefan Gaget (INSERM UMR 1283, CNRS UMR 8199, University of Lille, Lille, France) for technical assistance. We also thank Mathilde Boissel and Lijiao Ning (INSERM UMR 1283, CNRS UMR 8199, University of Lille, Lille, France) for help with the statistical analyses. The PROMIS data used for analysis described in this manuscript were obtained from the database of Genotypes and Phenotypes (dbGaP) through dbGaP accession number phs000917. The burden analysis was performed using data from the UK Biobank (Application #67575). We extend our gratitude to the patients and their families for their participation in the study.

Funding

This work was supported by funding from the UK Medical Research Council (MRC) MR/S026193/1 (S.S., A.M.S., Y.C.L.T., S.O.R., P.F., G.S.H.Y.) and the MRC Metabolic Diseases Unit (MC_UU_00014/1) (Y.C.L.T., S.O.R., G.S.H.Y.). Further support was provided by the National Center for Precision Diabetic Medicine – PreciDIAB (which is jointly supported by the French National Agency for Research (ANR-18-IBHU-0001), European Union (FEDER), Hauts-de-France Regional Council, European Metropolis of Lille (MEL) (A.B. and P.F.), European Union's Horizon Europe Research and Innovation Programme (OBELISK grant agreement 101080465 to A.B. and P.F.), France Génomique consortium (ANR-10-INBS-009 to A.B. and P.F.), European Research Council (OpiO 101043671 to A.B.) and Pakistan Academy of Sciences (M.A.).

Supplemental References:

1. Zorn S, von Schnurbein J, Schirmer M, Brandt S, and Wabitsch M. Measuring hyperphagia in patients with monogenic and syndromic obesity. *Appetite*. 2022;178:106161.
2. Key AP, and Dykens EM. Eye tracking as a marker of hyperphagia in Prader-Willi syndrome. *Developmental neuropsychology*. 2018;43(2):152-61.
3. Pujol J, Blanco-Hinojo L, Esteba-Castillo S, Caixàs A, Harrison BJ, Bueno M, et al. Anomalous basal ganglia connectivity and obsessive–compulsive behaviour in patients with Prader Willi syndrome. *Journal of Psychiatry and Neuroscience*. 2016;41(4):261-71.
4. Blanco-Hinojo L, Pujol J, Esteba-Castillo S, Martínez-Vilavella G, Giménez-Palop O, Gabau E, et al. Lack of response to disgusting food in the hypothalamus and related structures in Prader Willi syndrome. *NeuroImage: Clinical*. 2019;21:101662.
5. Jauregi J, Laurier V, Copet P, Tauber M, and Thuilleaux D. Behavioral profile of adults with Prader-Willi syndrome: correlations with individual and environmental variables. *Journal of Neurodevelopmental Disorders*. 2013;5:1-10.
6. Richards S, Aziz N, Bale S, Bick D, Das S, Gastier-Foster J, et al. Standards and guidelines for the interpretation of sequence variants: a joint consensus recommendation of the American College of Medical Genetics and Genomics and the Association for Molecular Pathology. *Genetics in medicine*. 2015;17(5):405-23.
7. Saeed S, Arslan M, Manzoor J, Din SM, Janjua QM, Ayesha H, et al. Genetic Causes of Severe Childhood Obesity: A Remarkably High Prevalence in an Inbred Population of Pakistan. *Diabetes*. 2020;69(7):1424-38.
8. Sun J, Zheng Y, and Hsu L. A unified mixed-effects model for rare-variant association in sequencing studies. *Genet Epidemiol*. 2013;37(4):334-44.
9. Saleheen D, Zaidi M, Rasheed A, Ahmad U, Hakeem A, Murtaza M, et al. The Pakistan Risk of Myocardial Infarction Study: a resource for the study of genetic, lifestyle and other determinants of myocardial infarction in South Asia. *European journal of epidemiology*. 2009;24:329-38.
10. Saeed S, Ning L, Badreddine A, Mirza MU, Boissel M, Khanam R, et al. Biallelic mutations in P4HTM cause syndromic obesity. *Diabetes*. 2023:db221017.
11. Lonsdale J, Thomas J, Salvatore M, Phillips R, Lo E, Shad S, et al. The genotype-tissue expression (GTEx) project. *Nature genetics*. 2013;45(6):580-5.
12. Steuernagel L, Lam BY, Klemm P, Dowsett GK, Bauder CA, Tadross JA, et al. HypoMap—a unified single-cell gene expression atlas of the murine hypothalamus. *Nature metabolism*. 2022:1-18.
13. Zhao Y, Chukanova M, Kentistou KA, Fairhurst-Hunter Z, Siegert AM, Jia RY, et al. Protein-truncating variants in BSN are associated with severe adult-onset obesity, type 2 diabetes and fatty liver disease. *Nature Genetics*. 2024;56(4):579-84.
14. Chen HJC, Mazzaferro S, Tian T, Mali I, and Merkle FT. Differentiation, Transcriptomic Profiling, and Calcium Imaging of Human Hypothalamic Neurons. *Current protocols*. 2023;3(6):e786.
15. Kirwan P, Jura M, and Merkle FT. Generation and characterization of functional human hypothalamic neurons. *Current protocols in neuroscience*. 2017;81(1):3.33. 1-3.. 24.

16. Yuan SH, Martin J, Elia J, Flippin J, Paramban RI, Hefferan MP, et al. Cell-surface marker signatures for the isolation of neural stem cells, glia and neurons derived from human pluripotent stem cells. *PloS one*. 2011;6(3):e17540.
17. Baldini L, Robert A, Charpentier B, and Labialle S. Phylogenetic and molecular analyses identify SNORD116 targets involved in the Prader–Willi syndrome. *Molecular biology and evolution*. 2022;39(1):msab348.
18. Dykens EM, Maxwell MA, Pantino E, Kossler R, and Roof E. Assessment of hyperphagia in Prader-Willi syndrome. *Obesity*. 2007;15(7):1816-26.
19. Holm VA, Cassidy SB, Butler MG, Hanchett JM, Greenswag LR, Whitman BY, et al. Prader-Willi syndrome: consensus diagnostic criteria. *Pediatrics*. 1993;91(2):398-402.
